# Whole-Genome Sequencing Reveals a New Genospecies of *Methylobacterium* sp. GXS13, Isolated from *Vitis vinifera* L. Xylem Sap

**DOI:** 10.1128/genomeA.01695-15

**Published:** 2016-02-04

**Authors:** Wan Xin Lai, Han Ming Gan, André O. Hudson, Michael A. Savka

**Affiliations:** a School of Science, Monash University Malaysia, Jalan Lagoon Selatan, Selangor, Malaysia; b Monash University Malaysia Genomics Facility, Monash University Malaysia, Jalan Lagoon Selatan, Selangor, Malaysia; c The Thomas H. Gosnell School of Life Sciences, College of Science, Rochester Institute of Technology, Rochester, New York, USA

## Abstract

The whole-genome sequence of a new genospecies of *Methylobacterium* sp., named GXS13 and isolated from grapevine xylem sap, is reported and demonstrates potential for methylotrophy, cytokinin synthesis, and cell wall modification. In addition, biosynthetic gene clusters were identified for cupriachelin, carotenoid, and acyl-homoserine lactone using the antiSMASH server.

## GENOME ANNOUNCEMENT

Members of the genus *Methylobacterium* are fastidious Gram-negative rods known for their methylotrophic metabolism, i.e., ability to utilize C1 compounds as their sole carbon and energy source. *Methylobacterium* species have been isolated from soil, leaf surface, grape xylem fluid, water, diseased tissue, and biochemical reagents (1–3).

Some *Methylobacterium* spp. are plant associated as during growth plants emit substantial amounts of methanol through the stomata (4). In some cases, *Methylobacterium* species also exhibited a symbiotic relationship with their plant host through the production of cytokinin that stimulates seed germination and plant development. Previously, we characterized and sequenced the whole genome of a grapevine xylem isolate, *Methylobacterium* sp. GXF4, leading to the identification of two *luxIR* homologs implicated in cell-to-cell communication and a unique β-galactosidase gene.

In the work reported here, we performed low-coverage whole-genome sequencing on morphologically dissimilar pink-pigmented bacterial isolates from the same grapevine xylem fluid and identified a genomically distinct isolate, strain GXS13, based on *in silico* genome-genome hybridization against *Methylobacterium* sp. GXF4 (5, 6).

Genomic DNA (gDNA) was extracted from strain GXS13 grown on potato dextrose agar medium for 5 days and prepped using the NEBNext Ultra DNA library prep kit (New England Biolabs, Ipswich, MA). The library was quantified and subsequently sequenced on the Illumina MiSeq (Illumina, San Diego, CA) at the Monash University Malaysia Genomics Facility. The raw reads were adapter trimmed with Trimmomatic 0.33 (7) and assembled using Spades 3.5.0 (8). The assembly contains 112 contigs with a total genome size of 5,805,293 bp (*N*_50_ of 109,000 bp; GC content of 68.50%; 85× coverage). Initial taxonomy assignment was performed using SpecI (9) and subsequently refined based on average nucleotide identity (ANI) analysis with JSpecies. Genome annotation based on PGAAP (10) led to the identification of 5,159 open reading frames (ORFs), 50 tRNAs, and 12 rRNAs.

SpecI assigned strain GXF13 to the genus *Methylobacterium*. A similarity search against *Methylobacterium* type strain gene sequences indicated that strain GXS13 is closely related to *Methylobacterium mesophilicum* DSM 1708T (16S rRNA and gyrB gene identities of 99.58% and 92.65%, respectively). ANI analysis shows that strain GXS13 has the highest score of 89.89% (as of October 2015) to *Methylobacterium* sp. GXF4 (6). Methanol oxidation genes were identified at contig 2 (*mxaFJGIRSACKLDEHB*), contig 3 (*mxcQE*), contig 1 (*mxbMD*), contig15 (*pqqA*), contig 1 (*pqqBCDE*), and contig 10 (*pqqFG*) (6, 11). Additionally, strain GXS13 also carries the *miaA* gene (contig 88) implicated in tRNA-mediated cytokinin synthesis via the isoprenylation of specific adenine in some tRNA (12, 13). Comparison of the strain GXS13 genome with publicly available *Methylobacterium* genomes identified a unique gene in strain GXS13 coding for CAZy family GT34 glycosyltransferase in contig 21 involved in the formation of a glycosidic bond between plant cell wall components, xyloglucans, and heteromannans (14–16). In addition, using the antibiotics and secondary metabolite analysis shell (antiSMASH) server (17, 18), cupriachelin, carotenoid, and acyl-homoserine lactone gene clusters were identified. The genomic potential for cytokinin synthesis and plant cell wall modification suggests the positive role of strain GXS13 in the growth and development of its host, grapevine.

### Nucleotide sequence accession numbers.

The nucleotide sequences have been deposited at DDBJ/EMBL/GenBank under accession number LKKO00000000. The BioProject number is PRJNA297388 and the BioSample number is SAMN04123207.
